# Immersive VR for upper-extremity rehabilitation in patients with neurological disorders: a scoping review

**DOI:** 10.1186/s12984-024-01367-0

**Published:** 2024-05-11

**Authors:** Matteo Ceradini, Elena Losanno, Silvestro Micera, Andrea Bandini, Silvia Orlandi

**Affiliations:** 1 The Biorobotics Institute and Department of Excellence in Robotics and AI, Scuola Superiore Sant’Anna, Pisa, Italy; 2 Modular Implantable Neuroprostheses (MINE) Laboratory, Università Vita-Salute San Raffaele & Scuola Superiore Sant’Anna, Milan, Italy; 3 Bertarelli Foundation Chair in Translational Neuroengineering, Center for Neuroprosthetics and Institute of Bioengineering, École Polytechnique Fédérale de Lausanne (EPFL), Lausanne, Switzerland; 4 Health Science Interdisciplinary Research Center, Scuola Superiore Sant’Anna, Pisa, Italy; 5 Department of Electrical, Electronic and Information Engineering “Guglielmo Marconi”, University of Bologna, Bologna, Italy; 6 IRCCS Istituto delle Scienze Neurologiche di Bologna, Bologna, Italy

**Keywords:** Upper extremity rehabilitation, Immersive virtual reality, Motor recovery, Novel rehabilitation strategies, Neurological disorders

## Abstract

**Background:**

Neurological disorders, such as stroke and chronic pain syndromes, profoundly impact independence and quality of life, especially when affecting upper extremity (UE) function. While conventional physical therapy has shown effectiveness in providing some neural recovery in affected individuals, there remains a need for improved interventions. Virtual reality (VR) has emerged as a promising technology-based approach for neurorehabilitation to make the patient’s experience more enjoyable. Among VR-based rehabilitation paradigms, those based on fully immersive systems with headsets have gained significant attention due to their potential to enhance patient’s engagement.

**Methods:**

This scoping review aims to investigate the current state of research on the use of immersive VR for UE rehabilitation in individuals with neurological diseases, highlighting benefits and limitations. We identified thirteen relevant studies through comprehensive searches in Scopus, PubMed, and IEEE Xplore databases. Eligible studies incorporated immersive VR for UE rehabilitation in patients with neurological disorders and evaluated participants’ neurological and motor functions before and after the intervention using clinical assessments.

**Results:**

Most of the included studies reported improvements in the participants rehabilitation outcomes, suggesting that immersive VR represents a valuable tool for UE rehabilitation in individuals with neurological disorders. In addition, immersive VR-based interventions hold the potential for personalized and intensive training within a telerehabilitation framework. However, further studies with better design are needed for true comparison with traditional therapy. Also, the potential side effects associated with VR head-mounted displays, such as dizziness and nausea, warrant careful consideration in the development and implementation of VR-based rehabilitation programs.

**Conclusion:**

This review provides valuable insights into the application of immersive VR in UE rehabilitation, offering the foundation for future research and clinical practice. By leveraging immersive VR’s potential, researchers and rehabilitation specialists can design more tailored and patient-centric rehabilitation strategies, ultimately improving the functional outcome and enhancing the quality of life of individuals with neurological diseases.

**Supplementary Information:**

The online version contains supplementary material available at 10.1186/s12984-024-01367-0.

## Background

Neurological disorders affect millions of people around the world. Stroke alone accounts for over 110 million cases, as reported by the World Health Organization [1]. Spinal cord injury, pain syndromes, multiple sclerosis, and many other diseases also affect a substantial number of people. These diseases not only have a physical impact, but also affect people's independence, well-being, and overall quality of life [2–4]. The global prevalence of neurological disorders and their profound impact underscore the urgent need for effective rehabilitation strategies to promote neurological recovery and improve the lives of those affected.

Neurological disorders often lead to impairments of the upper limbs, which are essential for performing everyday activities. To recover the use of their arms, patients often undergo conventional interventions, such as physical or occupational therapy [5–7]. Conventional physiotherapy for stroke survivors is often paired with technology-based interventions such as electromyographic biofeedback, electrostimulation, repetitive task training, and robotics [8]. Pain-related syndromes typically involve common interventions like mirror therapy, motor imagery, cognitive-behavioral therapies, and pharmacological treatments [9]. In the case of multiple sclerosis, commonly adopted interventions include robot-based training, home-based motor training, and electrical nerve stimulations [10]. Although these methods are effective in improving the outcomes with respect to conventional therapies alone [5, 8, 11], they also have limitations, such as low repeatability, high cost, and low engagement [12, 13]. Therefore, it is crucial to explore innovative technology-based approaches that can mitigate these limitations while improving rehabilitation outcomes [14].

Virtual reality (VR) is an end user human–computer interface technology that involves real-time simulation and interaction [15]. VR offers the possibility of engaging participants in multiple and personalized activities in which they can interact with virtual objects in real-time through multiple sensory modalities [16]. Immersive VR is an advanced form of VR that involves the use of head-mounted displays (HMDs) with high-resolution displays and spatial tracking systems to immerse users in a 3D virtual world that can be visually and audibly realistic. This combination of hardware and software allows users to engage with virtual objects and environments as though they were tangible realities [17]. The HMD includes a stereoscopic display that presents a different image to each eye, creating a sense of depth and immersion. Motion-tracking sensors detect the users' movements, allowing them to look around and interact naturally with the virtual environment.

In the context of rehabilitation, immersive VR is used as a tool to engage patients in virtual activities and therapeutic exercises specifically designed to promote their neurological recovery [18].

Currently, there is a limited understanding of the effectiveness, potential challenges, and facilitators associated with the use of immersive VR for upper limb rehabilitation across diverse neurological conditions. This scoping review aims to address this research gap by examining the characteristics and clinical outcomes of studies focusing on rehabilitation through immersive VR. Our analysis encompasses a comprehensive review of existing studies that utilize immersive VR for upper-limb rehabilitation in individuals with neurological disorders. Various aspects, including study type and design, population characteristics, neurological conditions, types of tasks employed, and rehabilitation outcomes, were thoroughly explored. The scope of our analysis extended beyond the intervention itself, encompassing specific details about the VR setup to provide a detailed account of the technical aspects. Additionally, we assessed potential side effects associated with the use of HMDs, an integral component of the immersive VR experience that requires careful consideration.

## Materials and methods

### Study design

This scoping review was performed in accordance with the Arksey and O’Malley scoping review methodology [19], which involves five mandatory steps, namely: (1) defining the research question; (2) identifying relevant studies; (3) study selection; (4) data extraction; and (5) analyzing the data, summarizing and reporting the results. An optional sixth step involves discussions among the raters. The available literature was summarized using the Preferred Reporting Items for Systematic Reviews and Meta-Analyses for Scoping Reviews (PRISMA-ScR) guidelines [20].

We based the study questions on the Sample, Phenomenon of Interest, Design, Evaluation, Research type (SPIDER) approach, commonly used for qualitative, quantitative and mixed methods reviews [21]:

- Sample: patients with peripheral or central neurological diseases.
- Phenomenon of interest: immersive VR-based treatment.
- Design: quantitative studies.
- Evaluation: barriers and facilitators of VR – HMD-based rehabilitation.
- Research type: primary studies and literature including only journal articles.

### Research question and eligibility criteria

Based on the objective of this scoping review, we identified the following research question: “What is known about the use of immersive VR for upper-extremity rehabilitation in patients with neurological diseases?”.

Based on the identified research question, we included the articles that met the following eligibility criteria:

- Studies on rehabilitation of the UE.
- Individuals affected by neurological disorders.
- Fully immersive VR (using HMD).

We excluded the articles that met the following exclusion criteria:

- Conference papers, expert opinions, editorials, and letters.
- Not English language.
- Not available full text articles.

To answer the research question, an additional inclusion criterion was applied to the full text of the eligible papers:

- Use of at least one clinical or kinematic outcome metric for evaluating the results of the interventions.

The clinical outcome metrics were considered valid if they were among the standard measures for clinical assessment of upper-limb function. Examples include the Fugl-Meyer Assessment-Upper Extremity (FMA-UE) [22], Modified Ashworth Scale (MAS) [23], Test of Upper Limb Apraxia (TULIA) [24], Box and Blocks Test (BBT) [25], and Action Research Arm Test (ARAT) [26]. The kinematic outcome metrics were considered valid if they provided an objective assessment of upper-limb function. Examples include the Range of Motion (ROM) and kinetic measures such as force and torque.

### Search strategy

To identify potentially relevant studies to be included in this scoping review, a systematic search—from inception to end of December 2023—was conducted on the following bibliographic databases: Scopus, PubMed, and IEEE Xplore.

Based on the identified research question, the systematic search was conducted using specific key terms, in Medical Subject Headings (MeSH) where applicable, to search through titles, abstracts, and keywords. The full search strategy is described in detail in Additional file 1: Table S1. The selected key terms were Head-Mounted Display, Virtual Reality, Upper limb, and Rehabilitation.

### Data management and extraction

Data extracted from the articles obtained through the search process described above were downloaded and organized using Microsoft Excel. Duplicates were removed. All titles and abstracts were first independently screened for relevance by MC and EL using the inclusion and exclusion criteria described above. No instances of conflicting opinions came up during the screening phase that necessitated the involvement of another author for resolution. Relevant full-text papers were then further screened for final inclusion by MC and EL using the full-text inclusion criterion described above. From each article, one reviewer (M.C.) extracted the data pertaining to the information needed to answer the research question. The extracted data concerned the general characteristics of the study (i.e., participants’ neurological condition, time since injury, sample size, age of the population, participants’ previous experience with VR, aims of the work, study type, and study design), experimental protocol (i.e., type of task, session duration, participants pre-training, experiment duration, recorded physiological signals, and outcome measures), virtual environment (i.e., HMD device, control modality, custom-built or commercial virtual environment, game engine, and explicit use of the audio), and assessment of cybersickness (i.e., questionnaires and other types of assessment).

One reviewer (MC) conducted a thematic analysis to identify the factors acting as barriers and facilitators in the adoption of immersive VR for rehabilitation according to the authors of each study. This analysis relied on the primary results and discussions chapters of each article, aiming to extract and retrieve the key points emphasized by the respective authors. Initially, key points representing the obstacles, challenges, and limitations were identified and subsequently condensed into one or two representative words. Subsequently, a statistic for each keyword was built by counting their frequency of occurrence across all the papers. Lastly, to facilitate discussion and categorization, we organized these terms into three clusters: technology, training and usability.

## Results

### Overview of the included studies

**Fig. 1:**
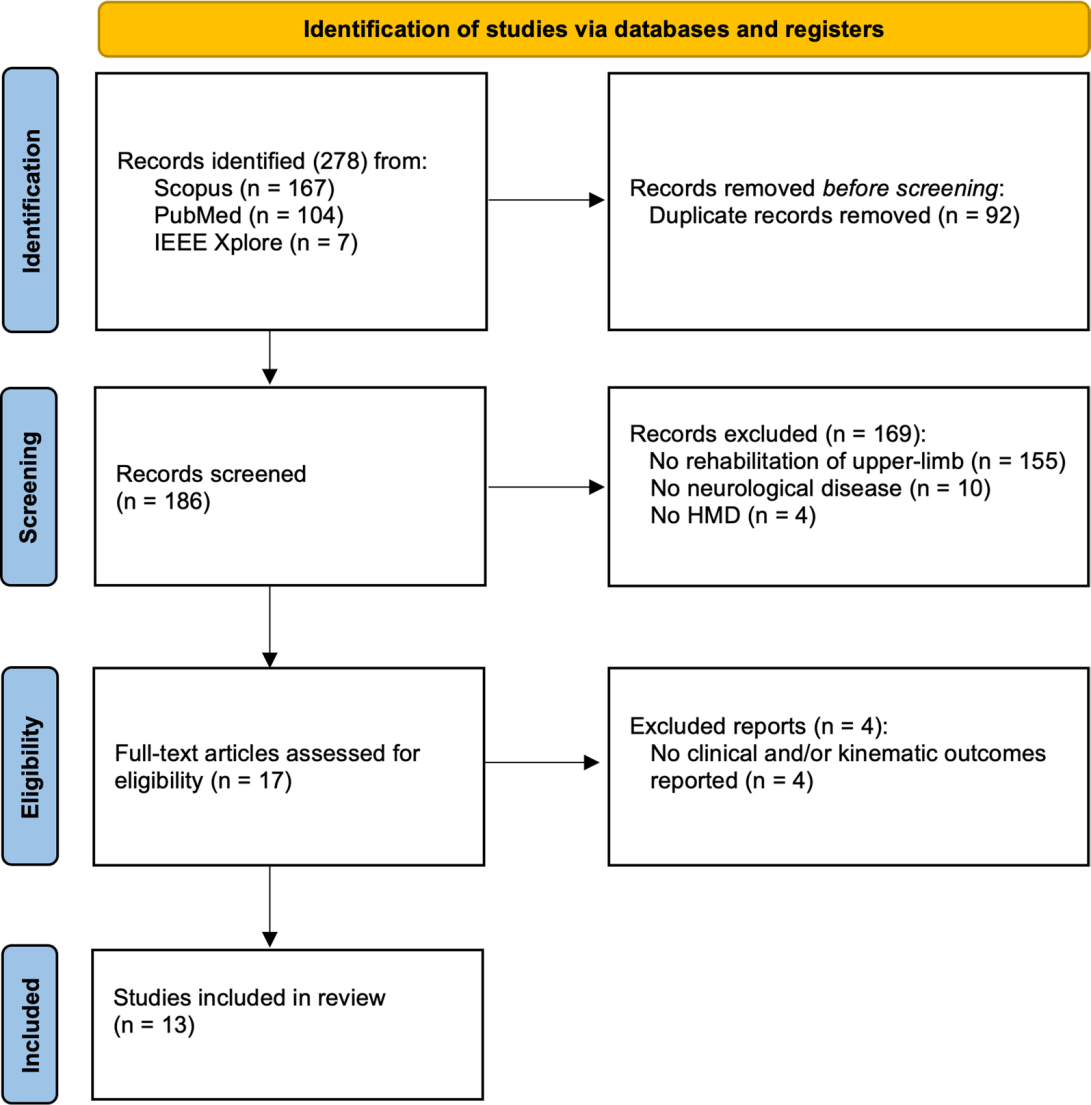
PRISMA flow diagram of scoping review results

The search yielded 309 articles from Scopus (n = 181), PubMed (n = 117), and IEEE Xplore (n = 11). After removing 96 duplicates, a total of 213 articles were screened by title and abstract and 195 were subsequently excluded, showing an almost perfect level of agreement between two authors M.C. and E.L. (Cohen’s Kappa coefficient = 0.914 [27]). The full text of 18 articles was evaluated for eligibility and 5 articles were eventually excluded because they did not meet the inclusion criteria for full text described in the Methods section (Cohen’s Kappa = 1.0 between MC and EL). Thus, the final synthesis included a total of 13 records (Fig. 1).

A comprehensive summary of key information extracted from the studies, including pathology, time since injury, sample size, population age, participants’ prior experience with VR, research aims, and study type and design is provided in Table 1. The analysis of these results reveals a notable trend: the use of immersive VR for upper-limb rehabilitation has emerged relatively recently, with the oldest paper included in this review published in 2016.

**Table 1:** Participants, study aims and design

Study	Pathology	Time since injury, mean (SD)	Sample size (women)	Population age [years] (mean)	Previous experience with VR	Aims of the work	Study type	Study design
Kamm et al. 2023 [43]	Multiple sclerosis	15.38 years (9.95)	11 (7)	n.r. (49)	No	Evaluating the feasibility, usability, and patient engagement/satisfaction of a home-based immersive virtual reality (VR) headset-based dexterity training in persons with multiple sclerosis	Observational	Uncontrolled non-randomized non-blinded
Heinrich et al. 2022 [30]	Stroke	63.36 days (58.22)	11 (4)	51–75 (62.1)	No	Proving that immersive VR mirror therapy is equivalent to traditional mirror therapy	Interventional	Uncontrolled non-randomized non-blinded equivalence
Park et al. 2021 [31]	Stroke (Ideomotor apraxia)	n.r	1 (0)	56	n.r	Comparing the therapeutic potencies of VR, conventional occupational therapy, and augmented reality	Case study	Exploratory research
Won et al. 2021 [39]	Complex regional pain syndrome	5 years	9 (6)	19–60 (44)	n.r	Investigating the use of VR in the treatment of patients with unilateral upper limb CRPS	Pilot study	Uncontrolled non-randomized non-blinded
Erhardsson et al. 2020 [32]	Stroke	2.51 years (1.96)	7 (2)	48–74 (60.6)	n.r	Investigating HMD-VR potential for chronic stroke upper-extremity rehabilitation and identifying suitable games, beneficiaries, and measures for evaluating the outcomes	Interventional	Uncontrolled non-randomized non-blinded case study
Marin-Pardo et al. 2020 [33]	Stroke	3.17 years (1.03)	4 (1)	42–66 (56.3)	n.r	Testing an EMG-based HMD-VR neurofeedback rehabilitation system to enhance muscle activity and reduce co-contractions in stroke patients	Pilot study	Uncontrolled non-randomized non-blinded
Lee et al. 2020 [34]	Stroke	3 years (5.16)	12 (5)	19–70 (40.2)	n.r	Investigating the feasibility of an immersive VR-based rehabilitation program for upper limb function in stroke patients	Interventional	Feasibility study uncontrolled non-randomized non-blinded
Weber et al. 2019 [35]	Stroke	6.83 years (4.21)	10 (4)	25–67 (54.1)	n.r	Testing the feasibility and evidence of the efficacy of immersive VR-based mirror therapy for upper limb function after stroke	Interventional	Uncontrolled non-randomized non-blinded
Vourvopoulos et al. 2019 [36]	Stroke	10 months	1 (0)	60	No	Testing the efficacy of an EEG-based BCI-VR system using a MI rehabilitation paradigm after a stroke	Case study	Exploratory research
Osumi et al. 2019 [40]	Phantom limb pain	11.63 years (10.14)	19 (5)	23–71 (48.1)	n.r	Revealing the relationship between VR effects and PLP characteristics using an immersive VR-based rehabilitation protocol	Interventional	Uncontrolled non-randomized non-blinded
Huang et al. 2018 [37]	Stroke	2.19 months (1.13)	8 (5)	44–79 (69)	n.r	Combining adaptive assist-as-needed control and immersive VR into a system for fine hand motion rehabilitation	Interventional	Uncontrolled non-randomized non-blinded
Chau et al. 2017 [41]	Phantom limb pain	5 months	1 (0)	49	No	Treating PLP with therapy in a custom immersive VR environment	Case study	Exploratory research
Osumi et al. 2017 [42]	Phantom limb pain	20.13 years (10.48)	8 (1)	43–64 (52.1)	n.r	Investigating an immersive VR-based short-term neurorehabilitation program for restoring voluntary movement representations and alleviating PLP	Interventional	Uncontrolled non-randomized non-blinded

*n.r.* not reported, *VR* virtual reality, *HMD* head-mounted display, *PLP* phantom limb pain, *CRPS* complex regional pain syndrome, *EEG* electroencephalography, *BCI* brain–computer interface, *MI* motor imagery

### Key characteristics of selected studies

#### Type of diseases

**Fig. 2:**
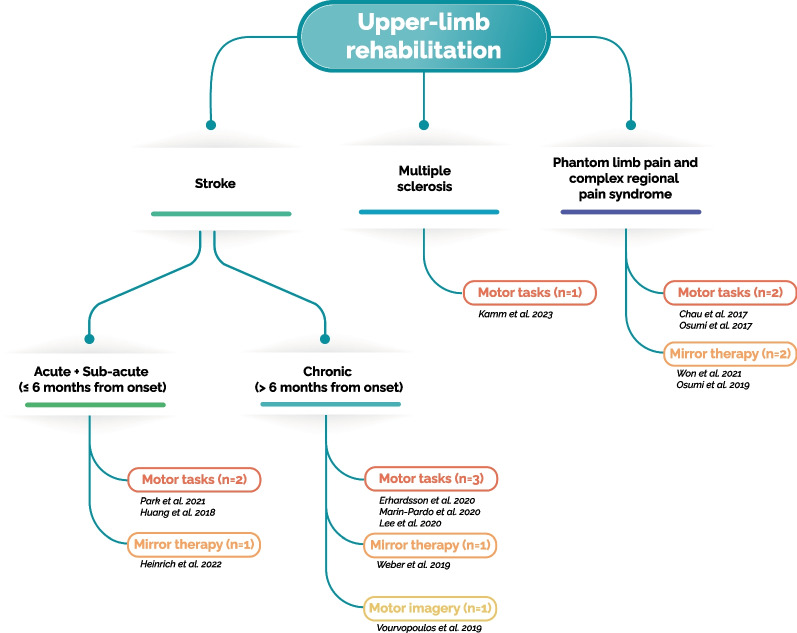
Studies taxonomy structured per pathologies and tasks

Given the global prevalence of stroke [28], stroke survivors have naturally emerged as primary candidates for immersive VR-based rehabilitation programs (Fig. 2). Symptoms of stroke vary depending on the damaged brain area, but in more than 80% of the cases, patients have upper limb impairments [29], including weakness or paralysis, decreased range of motion, spasticity, coordination problems, sensory alteration, and impaired fine motor skills [30]. All the analyzed studies focusing on stroke survivors aimed at improving their upper-limb motor functions (n = 8) [30–37]. Moreover, stroke conditions can be classified based on the time elapsed from the lesion between the acute stage (1–7 days), subacute stage (≤ 6 months) and chronic stage (> 6 months) [38]. We found that the studies selected in this scoping review were almost equally distributed among two groups, with three focusing on subacute patients [30, 31, 37] and the other five focusing on chronic patients [32–36]. The presence of studies that used immersive VR in acute and subacute phases of stroke rehabilitation reflects the clinical priority given to interventions during these vital early stages, emphasizing the importance of acting as soon as possible [8, 11].

Other two types of diseases for which studies using immersive VR for upper-limb rehabilitations has been used are conditions associated with pain (n = 4) [39–42], such as phantom limb pain and complex regional pain syndromes, and multiple sclerosis (n = 1) [43].

#### Type of virtual tasks

The studies identified in this scoping review can be further categorized based on the virtual rehabilitation tasks performed by the participants (Table 2). Three main types of tasks were used: mirror therapy, motor tasks, and motor imagery.

**Table 2:** Experimental protocols

Study	Type of task	Single session duration	Participant pre-training	Experiment duration	Physiological signals recorded	Rehabilitation metrics
Kamm et al. 2023 [43]	Object manipulation, fingers and wrist movements	20 min	No	2 Weeks: 10 sessions	No	Nine Hole Peg Test (9HPT) [58], Coin Rotation Task (CRT) [59], handheld JAMAR dynamometer [60], Multiple Sclerosis Impact Scale (MSIS-29) [61]
Heinrich et al. 2022 [30]	Mirror therapy	Mean 13.39 min (SD = 3.03)	No	3 Interventions, 3 sessions per intervention	No	Fugl-Meyer Assessment-Upper Extremity (FMA-UE) [22], Modified Rankin Scale (MRS) [62], Modified Ashworth Scale (MAS) [63], National Institutes of Health-Stroke Scale (NIH-SS) [64], Rivermead Assessment of Somatosensory Performance (RASP) [65]
Park et al. 2021 [31]	Reach and grasp	20 min	No	4 Weeks: 20 sessions	No	Fugl-Meyer Assessment (FMA) [48], Test of Upper Limb Apraxia (TULIA) [24], Korean Version of the Modified Barthel Index (K-MBI) [66]
Won et al. 2021 [39]	Mirror therapy (hitting targets)	n.r	No	From 3 to 5 sessions	No	PROMIS [67]
Erhardsson et al. 2020 [32]	Commercial game involving hand movement	As much as possible	No	Baseline (5 weeks: 5 sessions) + intervention (10 weeks: 10 sessions) + 6-month follow-up (1 session)	No	Fugl-Meyer Assessment-Upper Extremity (FMA-UE) [22], Action Research Arm Test (ARAT) [26], Box and Blocks Test (BBT) [25], Ashworth Scale [23], Saltin-Grimby Physical Activity Level Scale (SGPALS) [68]
Marin-Pardo et al. 2020 [33]	Movement attempt for pushing an object	1 h	Yes (2 sessions)	2 Weeks: 7 sessions	EMG (all sessions), EEG (first and last session—for corticomuscular coherence evaluation)	Fugl-Meyer Assessment-Upper Extremity (FMA-UE) [22], Action Research Arm Test (ARAT) [26], Montreal cognitive assessment (MOCA) [69], Sixteen-question stroke impact scale (SIS) [70]
Lee et al. 2020 [34]	Motor task from commercial applications	30 min	Yes	10 Sessions—2/3 sessions per week	No	Modified Barthel Index (MBI) [71], Action Research Arm Test (ARAT) [26], Box and Blocks Test (BBT) [25]
Weber et al. 2019 [35]	Mirror therapy	30 min	No	4 Weeks: 12 sessions	No	Fugl-Meyer Assessment-Upper Extremity (FMA-UE) [22], Action Research Arm Test (ARAT) [26], Star Cancellation Test [72]
Vourvopoulos et al. 2019 [36]	Motor Imagery	15 min	Yes (for BCI)	3 Weeks: 10 sessions	EEG, fMRI (3 sessions: pre, post, follow-up)	Fugl-Meyer Assessment-Upper Extremity (FMA-UE) [22], Ashworth Scale [23], Montreal cognitive assessment (MOCA) [69], Sixteen-question stroke impact scale (SIS) [70]
Osumi et al. 2019 [40]	Mirror therapy	20 min	No	1 Session	No	Short-Form McGill Pain Questionnaire—Japanese version (SF-MPQ) [50]
Huang et al. 2018 [37]	Objects manipulation	5 min	Yes	6 Weeks: 18 sessions, 3 sessions per week	No	Fugl-Meyer Assessment (FMA) [48], Motor Assessment Scale (MAS) [73]
Chau et al. 2017 [41]	Interaction with a virtual kitchen	45 min	No	5 Weeks: 5 sessions	EMG	Short-Form McGill Pain Questionnaire (SF-MPQ) [49], Wong-Baker FACES measurements [74]
Osumi et al. 2017 [42]	Reach and touch	10 min	No	1 Session	No	Short-Form McGill Pain Questionnaire (SF-MPQ) [49]

*VR* virtual reality, *EEG* electroencephalography, *EMG* electromyography, *fMRI* functional magnetic resonance imaging, *BCI* brain computer interface

Mirror therapy is a rehabilitation technique that traditionally uses a mirror to create a visual illusion of normal limb movement [44]. By reflecting the unaffected limb, it makes the brain perceive that the affected limb is functioning properly. VR mirror therapy is a variant of traditional mirror therapy in which patients see a virtual representation of their affected limb moving in synchrony with the actual movement of the unaffected limb [30, 35, 39, 40]. By having the illusion of the correct functioning of the affected limb, the brain receives positive visual feedback that can help alleviate pain, reduce swelling, and improve motor function and coordination. Mirror therapy takes advantage of the brain's neuroplasticity and promotes the rewiring of neural pathways and the reintegration of sensory and motor functions [41].

Motor rehabilitation tasks aim to improve motor control, coordination, and functional abilities by actively engaging the affected limb in goal-directed actions. In the selected studies, motor tasks involved using the affected limb to reach and grasp objects freely moving in the virtual environment [31, 42], interacting with a virtual kitchen [37, 41], manipulating objects [37], or other arm movements (e.g., wrist or fingers extension) [32–34, 43].

Another adopted approach was motor imagery [36]. Motor imagery involves mental rehearsal or imagination of specific movements without physically executing them. It is a cognitive technique often employed in stroke rehabilitation to promote motor recovery [45]. Patients imagine performing specific actions with their affected limb and the imagined movements are translated into motor commands from brain signals, usually recorded with electroencephalography (EEG) [46, 47]. In the study selected in this review, the participants were asked to row a boat by imagining moving either the left or the right hand [36].

#### Prior VR experience

We found that any of the included studies contained information regarding participants' prior experience with VR. This gap is likely due to the relatively recent emergence of immersive VR for rehabilitation purposes. Consequently, for the authors, it is improbable to encounter participants who have previously been exposed to this paradigm.

#### Type of clinical assessment

The selected studies included in our review show a considerable diversity in the implemented clinical assessments and outcome measures (Table 2). Among the studies on stroke patients, the most utilized assessment was the Fugl-Meyer Assessment (FMA) [48] or Fugl-Meyer Assessment-Upper Extremity (FMA-UE) [22], employed in seven out of eight studies [30–33, 35–37]. In addition, the Action Research Arm Test [26] was used in four out of eight studies [32–35]. Regarding the studies on phantom limb pain and complex regional pain syndrome, the most prevalent assessment was the Short-Form McGill Pain Questionnaire [49], which, along with its Japanese version [50], was used in three out of four studies [40–42].

### Characteristics of studies on stroke survivors

Among the studies in acute and subacute stroke patients, rehabilitation tasks are quite balanced (Fig. 2). Specifically, mirror therapy was used in one study [30], and motor tasks in two studies [31, 37]. Conversely, the studies in chronic patients mainly used motor tasks (n = 3) [32–34] and less, mirror therapy (n = 1) [35] and motor imagery (n = 1) [36].

In terms of homogeneity regarding clinical and demographic characteristics within subgroups, the three studies within the acute plus subacute stroke group focusing on motor tasks exhibit similar age ranges (Table 1): Heinrich et al. [30] reported participants’ ages ranging from 51 to 75 with a mean of 62 years, the single subject in the case study by Park et al. [31] was 56 years old, and Huang et al. 2018 [37] reported participants’ ages ranging from 44 to 79 years with a mean age of 69 years. The time since injury reported was similar for two studies: Heinrich et al. [30] (mean: 2.04 months; std: 1.87), and Huang et al. [37] (mean: 2.19 months; std: 1.13), while it cannot be compared as it was not reported by Park et al. [31]. The three studies on chronic stroke patients in the motor task subgroup show comparable clinical profiles in terms of time since injury: Erhardsson et al. [32] (mean: 2.51 years; std: 1.96), Marin Pardo et al. [33] (mean: 3.17 years; std: 1.03), and Lee et al. 2020 [34] (range: 3 years; std: 5.16). Furthermore, the age profiles are consistent between Erhardsson et al. [32] (range: 48–74 years; mean: 60.6) and Marin Pardo et al. [33] (range: 42–66 years; mean: 56.3). Only Lee et al. [34] have a population with a wider age range, starting from a lower minimum (range: 19–70 years; mean: 40.2).

As regards, instead, the comparison between studies in acute plus sub-acute and chronic stroke patients, there are some differences including the duration of a single session and the total number of sessions conducted (Table 2). For studies with participants in the sub-acute post-stroke stage [30, 31, 37], who are more sensitive to the intensity and duration of exercises because of their fragile condition, the average session duration was 13 ± 6 (mean ± std) minutes, and the average number of sessions was 14 ± 6. In contrast, in the studies involving chronic stroke patients [32–36], longer experimental sessions were performed, lasting 40 ± 17 min on average. In this case, the total number of sessions was lower, 11 ± 4 sessions on average. These findings suggest that the duration and repetition of the experimental protocol may vary based on the stroke phase, resulting in personalized approaches tailored to individual patient’s conditions and needs. However, it is important to note that the lack of studies with a direct comparison between interventions for both sub-acute and chronic stroke patients, makes it challenging to conclusively determine whether the resulting difference is primarily influenced by participants stroke condition or by the implementation of diverse tasks across studies.

### Characteristics of studies on patients with other neurological conditions

The types of rehabilitation tasks are almost evenly distributed among the studies in patients with phantom limb pain and complex regional pain syndrome. Specifically, two studies used mirror therapy [39, 40], and the other three employed motor tasks [41–43] (Fig. 2). Furthermore, there was significant variability in the time since injury, 9 ± 8 years on average across studies, with a minimum of 5 months and a maximum of 20 years. The average number of sessions performed was 3 ± 2, with each session lasting 25 ± 18 min. The only study in patients with multiple sclerosis recruited participants 15.38 ± 9.95 years post-injury, who performed ten 20-min sessions of VR-based motor tasks.

Regarding the homogeneity within subgroups, the two studies within the group of phantom limb pain and complex regional pain syndrome on motor tasks recruited quite different populations (Table 1). The single subject in the study by Chau et al. [41] was recruited 5 months after injury, whereas the participants of the study by Osumi et al. [42] was recruited after an average of 20.13 years (std: 10.48). The age of the two populations, instead, is comparable: Chau et al. [41] (49 years) and Osumi et al. [42] (range 43–64 years; mean: 52.1). On the other hand, the two studies on mirror therapy reflect similar characteristics in both the clinical and demographic domains: Won et al. [39] (mean time since injury: 5 years; age range: 19–60 years, std: 44 years) and Osumi et al. [40] (mean time since injury: 11.63 years, std: 10.14; age range: 23–71 years, std: 48.1 years).

### Geographic locations of the studies

**Fig. 3:**
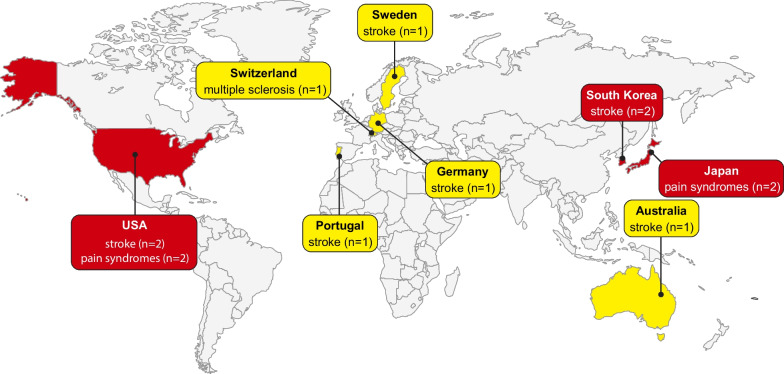
World map of the geographical distribution of the studies, organized by pathology

We analyzed the geographic distribution of the studies categorized by the type of neurological condition (Fig. 3). We established the geographical location of each study by considering the clinical sites where the participants were recruited and where the studies were conducted.

In the case of studies focused on stroke, we observed that half of the studies were evenly distributed between the United States of America (n = 2) [33, 35] and South Korea (n = 2) [31, 34]. The remaining studies were conducted in various European countries, including Germany [30], Sweden [32], and Portugal [36], with one study conducted in Australia [37]. When examining studies related to upper-limb pain syndromes, we observed an equal distribution between the United States of America (n = 2) [39, 41] and Japan (n = 2) [40, 42]. Lastly, the single study on patients with multiple sclerosis was conducted in Switzerland [43].

### The virtual setup

**Fig. 4:**
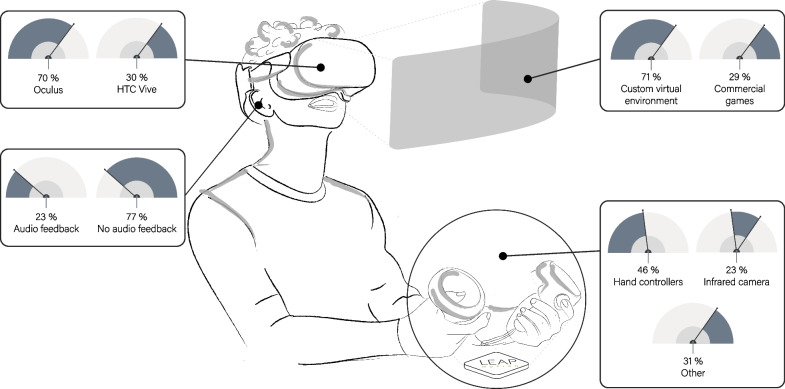
Immersive virtual reality setup. The percentage of studies that used different technologies, setups, audio feedback types, and controllers are reported

We reported the HMD, control modality, and VR environment selected by the authors of the studies included in this scoping review (Table 3; Fig. 4). As regards the choice of the HMD, the Oculus (Facebook Technologies, USA) was the preferred option and was used in 9 out of 13 studies [30, 33, 35–37, 39, 40, 42, 43]. The HTC Vive (HTC Corporation, Taiwan) was used in the 4 remaining studies [31, 32, 34, 41]. As for the control modality, the most popular option (n = 6) [32, 34, 35, 39, 41, 43] included hand controllers for tracking the position of the hands in space. However, holding a hand controller can be challenging or even impossible for individuals affected by hand motor impairment. To solve this issue, the authors of studies [32, 35, 41] employed bands or straps to secure the controller to the participants' arms. Alternatively, three studies implemented hand or finger tracking using the Leap Motion (Ultraleap, USA) infrared camera [30, 40, 42], and one study [43] reported the usage of the markerless tracking included in the Oculus Quest 2 system. Markerless tracking methods offer a significant advantage by eliminating the need for the subjects to hold or wear controllers, allowing them to move without constraints. At the same time, markerless systems may exhibit reduced robustness when dealing with freely moving upper arms in a large space [30]. The other control solutions adopted were electromyographic (EMG) signals of arm muscles [33], EEG signals during motor imagery [36], and a robotic device that tracked the finger’s position and force while assisting the participant’s movements [37].

**Table 3:** Virtual setup

Study	HMD device	Control modality	Custom virtual env	Game engine	Explicit audio use
Kamm et al. 2023 [43]	Oculus Quest 2	Hand controllers	Yes	n.r	No
Heinrich et al. 2022 [30]	Oculus Rift CV1	Infrared camera (Leap Motion) hand/fingers tracking	Yes	Unity	No
Park et al. 2021 [31]	HTC Vive	n.r	Yes	n.r	No
Won et al. 2021 [39]	Oculus Rift	Hand controllers (held in unaffected hand)	Yes	Unity	No
Erhardsson et al. 2020 [32]	HTC Vive	Hand controllers (attached with velcro straps)	No		No
Marin-Pardo et al. 2020 [33]	Oculus Rift CV1	Electromyography (EMG) controller	Yes	Unity	No
Lee et al. 2020 [34]	HTC Vive	Hand controllers (held in affected hand)	No		No
Weber et al. 2019 [35]	Oculus Rift	Hand controllers (held in unaffected hand and fastened to the wrist)	No		No
Vourvopoulos et al. 2019 [36]	Oculus Rift DK1	Electroencephalography (EEG) Brain Computer Interface (BCI)	Yes	Unity	Yes—auditory feedback (ambient + events sound)
Osumi et al. 2019 [40]	Oculus Rift	Infrared camera (Leap motion) hand/fingers tracking	Yes	n.r	No
Huang et al. 2018 [37]	Oculus Rift DK2	Hand rehabilitation robotic device	Yes	Unity	No
Chau et al. 2017 [41]	HTC Vive	Hand controllers (strapped to the upper-arm/residual forearm) + myoelectric (EMG) controller	No		Yes—to provide cues to the participants for guiding the experiment
Osumi et al. 2017 [42]	Oculus Rift	Infrared camera (Kinect and Leap Motion) for arm movements detection	Yes	Unity	Yes—auditory feedback

*n.r.* not reported, *EEG* electroencephalography, *EMG* electromyography, *fMRI* functional magnetic resonance imaging, *BCI* brain computer interface

In most of the cases (9 out of 13 studies) [30, 31, 33, 36, 37, 39, 40, 42, 43], the virtual environment was custom-built, and when reported, the Unity 3D game engine software (Unity Technologies, USA) was utilized. In the other cases, commercially available games were adopted [32, 34, 35, 41]. Regarding the use of audio during the interventions, only three studies explicitly reported it [36, 41, 42]. In all these cases, the audio was used for giving feedback and instructions to the participants.

### Rehabilitation outcomes

**Fig. 5:**
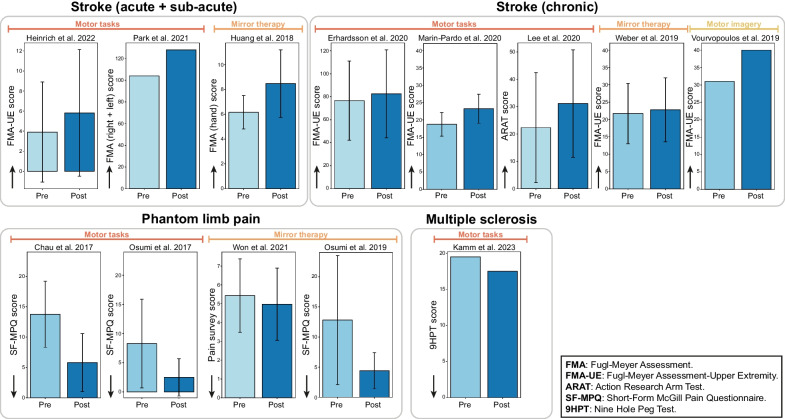
Rehabilitation outcomes. The figure illustrates the main rehabilitation outcome of each study, showing the pre- and post-intervention values of each study’s primary outcome metric on average (± standard deviation) across participants. The metric was chosen as either the one suggested by the authors, if available, or as the most used metric among the other studies. Studies are organized by neurological disorders, and the type of task implemented (motor tasks, mirror therapy, or motor imagery) is specified for each study. The black arrow in the bottom-left corner of each bar plot indicates the direction of the desired variation of the outcome metric. *FMA-UE* Fugl-Meyer Assessment-Upper Extremity, *ARAT* Action Research Arm Test, *SF-MPQ* short-form McGill Pain Questionnaire, *9HPT* 9-Hole Peg Test

The studies included in this scoping review reported motor or neurological recovery after fully immersive VR-based rehabilitation, as described in Table 4. In Fig. 5 we report the pre- and post-rehabilitation values of the primary outcome metric of each study; this metric was chosen either as the primary metric suggested by the authors, if available, or as the most used metric among the other studies included in this scoping review, to facilitate inter-study comparison. Almost all the studies, 12 out of 13 [30–37, 40–43], reported an improvement in at least one of the clinical metrics following the treatment for some or all the participants. Significant improvements were observed in 5 out of 8 studies focusing on stroke patients [30, 33, 34, 36, 37]. Additionally, 3 out of 4 studies on pain relief reported significant improvements in at least one of the outcome measures [40–42]. Finally, the single study on multiple sclerosis reported a significant improvement in one of the metrics evaluated [43]. This demonstrates that immersive VR is a valid approach to perform rehabilitation of various types of neurological disorders. Among the benefits reported, stroke survivors exhibited both physical improvements, such as increased strength, expanded range of motion, and enhanced coordination [31, 33, 36, 37], and cognitive improvements, such as better attention, memory, and executive functions [33, 36]. Finally, participants with stroke also reported functional benefits, such as increased independence in activities of daily living [31], improved mobility, and enhanced quality of life [33, 36]. Studies involving participants with pain-related syndromes generally reported significant pain alleviation.

**Table 4:** Study results

Study	Pathology	Main results reported
Kamm et al. 2023 [43]	Multiple sclerosis	Feasibility, usability, and patient engagement/satisfaction with the VR training were very high. The CRT for the dominant hand improved significantly after training (p = 0.03)
Heinrich et al. 2022 [30]	Stroke	Decreased motor impairment in the affected arm in 9/11 participants
Park et al. 2021 [31]	Stroke (Ideomotor apraxia)	TULIA score improved (from 121 to 161), DAL improved, MBI score improved (from 55 to 84), and other improvements in personal hygiene, bathing, toileting, dressing, stair climbing, ambulation, and transfer fields were reported
Won et al. 2021 [39]	Complex regional pain syndrome	No statistically significant differences over time on average or highest pain of the affected limb or body, or on physical activity, mood, or quality of sleep
Erhardsson et al. 2020 [32]	Stroke	Positive trend of improvement in all participants (independently from the impairment level). 3 participants improved in 3 to 5 outcome measures out of 6
Marin-Pardo et al. 2020 [33]	Stroke	At the group level, only the SIS-16 showed significant improvements, non-significant trends in that ARAT and FMA-UE. Range of active wrist extension improved for three participants. Trends of improved motor control were seen in 3/4 of participants after training, for both flexion and extension. Significant corticomuscular coherence was observed only during static holding of wrist extension and not during flexion
Lee et al. 2020 [34]	Stroke	5/9 participants, who complete the study, improved both in ARAT and BBT. BBT and MBI significantly improved after the training. Overall satisfaction was 6.3/7. Interest (6.4/7) and intent to continue training (6.4/7) items had the highest scores, whereas discomfort (4.9/7) had the lowest score
Weber et al. 2019 [35]	Stroke	A small improvement in FMA-UE and ARAT, but not statistically significant. SUS from 40 to 100 (AVG = 76)
Vourvopoulos et al. 2019 [36]	Stroke	FMA-UE improved significantly by 9 points after the intervention, followed by 4 points improvement in the follow-up. Muscle tonus was increased but did not interfere with range of motion. SIS showed a conspicuous increase in the strength domain. External Visual Imagery improved in post-intervention (and maintained in follow-up), Internal Visual Imagery improved in post-intervention (returned to same level in follow-up), and Kinesthetic Imagery stay to the same level
Osumi et al. 2019 [40]	Phantom limb pain	Distortion of the intact-hand line trajectory significantly increased after VR-MVF rehabilitation. SF-MPQ scores significantly decreased indicating that the VR-MVF rehabilitation successfully alleviated PLP. The scores of both questionnaire items regarding the sense of reality of the virtual phantom limb were significantly higher than 0
Huang et al. 2018 [37]	Stroke	FMA significantly improved in 4/8 participants, moderate improvements in 2/8, while only minor changes were obtained in 2/8. Increase in MAS score for all participants. 7/8 participants showed noticeable improvement in their range of motion
Chau et al. 2017 [41]	Phantom limb pain	All pain scales showed a statistically significant decrease in pain during each VR session. Significant subjective pain relief typically takes effect approximately 24 h after each VR session. On six-week follow-up, the participant reported that the pain was still present, but generally decreased in severity and was much better tolerated overall
Osumi et al. 2017 [42]	Phantom limb pain	SF-MPQ averaged across all participants significantly decreased. NRS pain scores decreased significantly. Sense of reality was significantly higher than zero

*CRT* coin rotation task, *TULIA* test of upper limb apraxia, *DAL* daily living activities, *MBI* Modified Barthel Index, *FMA-UE* Fugl-Meyer Assessment-Upper Extremity, *ARAT* Action Research Arm Test, *BBT* box and block test, *SUS* System Usability Scale, *SIS* Stroke Impact Scale, *SF-MPQ* Short-Form McGill Pain Questionnaire, *MAS* Motor Assessment Scale, *NRS* Numerical Rating Scale of PLP intensity, *AVG* average

## Barriers and facilitators

**Fig. 6:**
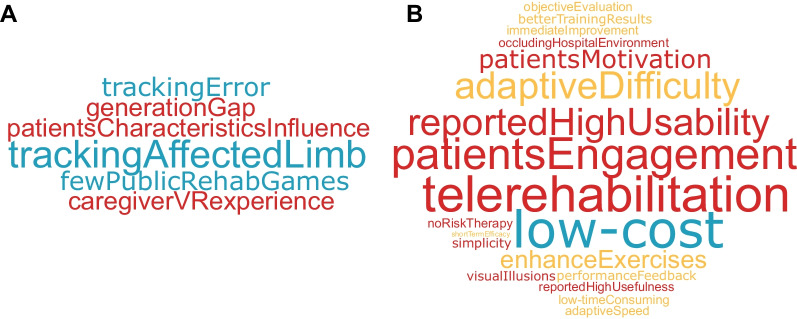
Barriers (**A**) and facilitators (**B**) word clouds. Keywords are characterized by font size and color: (i) larger font sizes indicate a higher frequency of a keyword’s appearance in the studies, (ii) the font color represents the thematic area to which a keyword refers

Here we report the barriers and facilitators identified by the authors of the included studies related to the use of immersive VR for upper-limb rehabilitation (Fig. 6).

The principal issue reported by the authors was related to technology, in particular errors in tracking the affected limb [30, 32, 34, 39–42], and low availability of public rehabilitation “games” [32]. An additional aspect that was mentioned, although not consistently reported in all the studies, pertained to the usability of immersive VR. This includes aspects such as the impact of participants’ characteristics, including generational differences, and caregiver technical experience on the study outcomes [32, 34, 42].

Among the facilitators of immersive VR for upper-limb rehabilitation, the most frequently reported factors related to ease of use, such as increased participants’ engagement and the possibility to conduct telerehabilitation sessions. Another factor mentioned is the possibility of providing therapies that include functional tasks even for individuals who are unable to physically perform them due to severe motor deficits. In addition, authors reported that hiding the hospital environment, which does not always have a positive impact on the therapy, may be helpful for patients [33].

Authors frequently discuss the economic aspect, highlighting that immersive VR technology has a reasonably affordable cost [35], typically ranging approximately between 300$ and 700$. However, the VR market is constantly evolving, potentially introducing newer headset versions at different price levels since the time this paper was written. Several authors also highlighted aspects regarding the flexibility of the training based on immersive VR, such as the possibility of continuously and easily adapting the difficulty of the exercises to the patient's clinical condition and rehabilitation status [30, 32, 33].

### Immersive VR side-effects

Immersive VR is commonly associated with a phenomenon known as cybersickness [51]. Cybersickness refers to unpleasant and negative effects that individuals may experience when using immersive VR. It is characterized by symptoms like motion sickness, including nausea, dizziness, disorientation, headache, sweating, and general discomfort [52]. The manifestations of cybersickness differ from person to person, with some people experiencing mild discomfort or disorientation and others encountering more severe symptoms that could impede their ability to use VR or force them to interrupt the VR experience. These symptoms can manifest during or after VR use and may persist for different periods of time [53].

Only 4 of the analysed 13 studies [30, 33, 35, 39] included an assessment of cybersickness (Table 5). Three studies [30, 33, 39] assessed cybersickness using the Simulator Sickness Questionnaire (SSQ) [54], whereas one study [35] employed the Motion Sickness Susceptibility Questionnaire Short Form (MSSQ-Short) [55]. None of the four articles reported significant cybersickness symptoms in the participants. Among the remaining studies, only three of them discussed the cybersickness issue [31, 32, 34]. However, none of them reported adverse effects or problems related to cybersickness in the participants.

**Table 5:** Cybersickness

Study	Cybersickness questionnaires	Cybersickness evaluation
Kamm et al. 2023 [43]	No	n.r
Heinrich et al. 2022 [30]	Simulator Sickness Questionnaire	No symptoms reported. SSQ results [mean (std)] = first intervention [2.36 (2.01)], second intervention = [2.45 (2.46)], third intervention [2.73 (2.34)]
Park et al. 2021 [31]	No	Symptoms only in the first session, but the participant resolved in a few minutes
Won et al. 2021 [39]	Simulator Sickness Questionnaire	7 Participants reported no cybersickness, with 2 participants rating their cybersickness as “slight.” No participants quit any trial because of its effects SSQ results not reported
Erhardsson et al. 2020 [32]	No	No serious adverse effects were observed during or after training. One participant felt slightly unsteady for a few hours post-training after the first sessions
Marin-Pardo et al. 2020 [33]	Simulator Sickness Questionnaire	No symptoms reported SSQ results [mean (std)] = first session [5.56 (5.27)], last session: [6.35 (2.59)]
Lee et al. 2020 [34]	No	No symptoms were reported in all patients
Weber et al. 2019 [35]	Motion Sickness Susceptibility Questionnaire Short Form	No symptoms reported. MSSQ-Short indicating little to no cybersickness MSSQ-Short results [mean] = first session [1], last session [1.6]
Vourvopoulos et al. 2019 [36]	No	n.r
Osumi et al. 2019 [40]	No	n.r
Huang et al. 2018 [37]	No	n.r
Chau et al. 2017 [41]	No	n.r
Osumi et al. 2017 [42]	No	n.r

*n.r.* not reported, *SSQ* simulator sickness questionnaire, *MSSQ-Short* motion sickness susceptibility questionnaire short form, *std* standard deviation

## Discussion

In this scoping review, we examined the use of immersive VR in upper-limb rehabilitation for individuals with neurological diseases. Thirteen studies were included and reviewed in terms of methodologies, side effects, and clinical outcomes. We provided an overview of the most adopted approaches in terms of types of tasks performed by the participants, rehabilitation metrics applied to evaluate the outcomes of the interventions and virtual environment setups. We found that this technology is relatively new in the field of neurorehabilitation and, up to now, the primary application has been on conditions such as stroke and pain-related syndromes, with no clear geographical pattern. In most of the cases the virtual environment was controlled using hand controllers and the authors preferred to build their own virtual environment for customization purposes. Remarkably, most of the studies documented enhancements in the evaluated rehabilitation metrics, with significant improvements observed in most cases.

In the following, we will discuss the barriers and facilitators highlighted by the authors, the advantages and disadvantages of customizing interventions, the lack of a standard in measuring and reporting the results, and finally on the limitations of the study design.

### Barriers and facilitators

The most recurrent barriers in the use of immersive VR for upper limb rehabilitation included motion tracking and limited adoption.

As previously mentioned, the use of controllers to track the affected limb can be problematic for some participants, even though a potential solution is to secure the controller to the arm using straps. On the other hand, the use of infrared and depth-sensing cameras, such as the Leap Motion, may introduce errors and limitations like high latency or insufficient accuracy [30]. Another solution, not exploited by any of the studies included in this review, is the usage of the Vive tracker when using the Vive HMD (HTC Corporation, Taiwan). The Vive tracker is a compact device that can effortlessly be attached to the participants' arms without impeding their movements. This device provides an accurate real-time tracking system [56] that can address the tracking errors and related issues. Additionally, both HTC and Oculus offer built-in hand-gesture recognition capabilities. However, none of the studies included in this scoping review exploited this functionality. Regarding the choice of the technology, most of the studies (Fig. 4) used the Oculus device [30, 33, 35–37, 39, 40, 42, 43], but the authors did not specify the rationale behind their choice. We speculate that one reason could be the price difference between the Oculus and HTC devices: the Oculus devices tend to be slightly cheaper than HTC devices (100$ difference approximately). Another contributing factor could be the ease of use and calibration of the Oculus devices: unlike HTC devices, Oculus systems do not require the placement of base stations in the room to track the headset and controllers, thus simplifying the setup procedures.

It has been reported [34] that the adoption of immersive VR may face obstacles due to generational differences and caregivers' limited technical experience. To address these challenges, it is essential to focus on training and education initiatives. Additionally, improving user-friendliness and developing more plug-and-play systems can enhance overall ease of use. Nevertheless, given the swift global surge in technology adoption, we anticipate that substantial advancements in these aspects are not only achievable but also imminent.

Regarding the facilitators, the immersive and interactive nature of VR environments, along with the element of fun and challenge, can motivate participants to exert more effort during rehabilitation exercises [30, 31, 33–35]. In addition, VR is suitable for telerehabilitation where patients perform the exercises from the convenience of their own home, guided, if needed, by voice instructions from the therapist. Telerehabilitation can reduce or even eliminate the need for patients to be physically present in the clinical facilities, reducing the inconvenience and costs associated with transfers. Furthermore, VR facilitates the rehabilitation of patients who find it difficult to undergo conventional therapy due to sever motor deficits [33]. Also, the availability of objective performance measures provides clinicians with a comprehensive picture of the patients’ progress and status [31, 37]. Finally, the flexibility of VR allows for the development of personalized interventions and their adaptation over time, ultimately increasing their effectiveness [30, 32, 33]. All these elements highlight the VR potential of allowing a patient-centric approach that can enhance the quality of care and improve functional outcomes. More effective rehabilitation can reduce the frequency of interventions and, combined with the affordability of VR technology, reduce overall healthcare costs [37].

### Customization of interventions

Immersive VR has shown promising potential for upper-limb pain relief therapy [40, 41]. A notable advantage of immersive VR is that it can integrate a variety of rehabilitation exercises that may not be implemented in traditional non-virtual environments, such as the creation of a more “sophisticated” immersive form of mirror-therapy [5, 57]. The virtual setting can overcome the constraints of the physical world, allowing innovative and tailored approaches to improve the effectiveness and engagement of the rehabilitation process. It is worth noting that most of the studies in our review created custom immersive VR environments. A custom-built environment offers the possibility to be tailored to the intended use-case in terms of therapists’ requirements and patients’ needs [30, 33]. However, the development of a 3D VR environment compatible with HMDs requires technical skills and expertise. To promote the wider adoption of immersive VR as a rehabilitation tool, there is a need for more commercial games specifically designed for this purpose.

### Lack of a standard: prior VR experience, cybersickness, and outcome measures

As outlined in the *Results* section, none of the studies within this scoping review discussed about the participants’ prior familiarity or experience with immersive VR technology. We recommend that authors of future studies consider the inclusion of this information in the participant data. We believe that participants’ prior experience with immersive VR can potentially introduce biases in the VR-based rehabilitation outcomes, particularly related to the usability and effectiveness.

Furthermore, most of the studies included in the analysis did not employ structured questionnaires for assessing the presence of cybersickness symptoms. This may contribute to underreporting [34] and, consequently, a deficiency in comprehensive evaluation. By collecting information on participants' prior experience with VR, implementing quantitative measures of cybersickness, and establishing standardized protocols tailored to specific pathologies, researchers and therapists could better assess and address the potential adverse effects of immersive VR-based rehabilitation.

Finally, as depicted in Fig. 5, we found a lack of standardization in reporting the primary clinical outcomes, even within the same study group (same neurological disease and rehabilitation task). Indeed, despite using the same metric (mostly FMA-UE for stroke studies and SF-MPQ for phantom limb pain studies), studies reported different ranges of values due to differences in the measurement methodology of the metric. This discrepancy makes it challenging to compare the results across different studies and approaches. A more standardized approach and guidelines for the choice of metrics for specific neurological disorders are needed in this field.

### Study limitations

Most of the problems reported by the authors are related to the research methodology, and in particular to study design [30, 32, 34–36, 40, 42] and limited sample size [30, 33–37, 39, 41, 43]. In particular, the authors emphasize that the number of participants plays a crucial role in determining the true effectiveness of immersive VR for upper-limb rehabilitation. Similarly, study design limitations, such as a limited number of interventions and the absence of randomized or controlled designs, pose challenges when evaluating the advantages of immersive VR for neurorehabilitation. These limitations extend to the possibly to compare these benefits with traditional rehabilitation therapies. In future studies, it is advisable to address these issues by incorporating a larger participant pool within randomized controlled trials.

### Future horizons

Envisaging the future of upper-limb rehabilitation through immersive VR interventions reveals several critical considerations highlighted in this scoping review.

Firstly, the establishment of standards and protocols is essential to ensure consistency among studies and facilitate quantitative comparisons of diverse interventions. Customizing VR interventions based on individual patient needs, including motor skills, disability levels, and personal preferences, is crucial for optimizing intervention effectiveness. Additionally, it is imperative to correlate quantifiable metrics extracted from gamified tasks with clinical outcomes to establish benchmarks for patients’ capacity during upper limb VR-rehab tasks. This step is vital for comparisons across different groups and tracking rehabilitation progress over time.

Secondly, understanding participant acceptability and adherence to VR interventions is paramount. The integration of qualitative and quantitative feedback obtained during gamified tasks should be supported by standardized protocols and questionnaires related to usability, sense of presence, and cybersickness. This aspect is crucial for the development of VR-based medical devices accepted in clinical settings, enabling manufacturers to create devices specifically tailored for clinical purposes.

Thirdly, expanding the scope of VR interventions to address various conditions requires assessing suitability across diverse age groups, disability levels, and clinical scenarios. Evaluating the long-term effects of VR interventions on upper limb functions is essential for a comprehensive assessment. Additionally, exploring new access technologies, such as brain-computer interfaces, holds promise in enhancing accessibility to VR-based rehabilitation, allowing individuals with motor disabilities to access more effective therapies by sending commands directly through the brain.

Considering the barriers and facilitators identified in this review, prioritizing the avoidance of potential adverse effects should be fundamental in VR rehabilitation protocols. A comprehensive collection of patient information regarding adverse effects from immersive VR usage is vital for targeted interventions and preventing negative impacts on patients.

Integrating VR into clinical care necessitates strategic planning, including examining effective strategies for incorporating VR rehabilitation into standard clinical practice. This involves healthcare provider training, ensuring patient accessibility, and active involvement of patients, caregivers, healthcare providers, technology developers, and researchers in a comprehensive and collaborative manner, utilizing participatory and user-centred design in the development of gamified tasks.

Collectively incorporated into future studies, these elements hold significant potential to profoundly shape the future landscape of rehabilitation therapies for upper-limb neurological disorders.

## Conclusion

This scoping review explored the use of immersive VR for upper limb rehabilitation in individuals with neurological diseases. Results indicate that immersive VR is a promising rehabilitation tool, especially for stroke survivors and individuals with phantom limb pain or complex regional pain syndrome. The studies used various tasks such as mirror therapy, motor tasks, and motor imagery. The VR-based rehabilitation program had positive neurological effects, such as increased strength, improved range of motion, higher coordination, and pain relief. Overall, immersive VR can offer personalized, engaging, and intensive rehabilitation to individuals with neurological disorders, thereby improving their recovery and quality of life. Nonetheless, further research is required to standardize participant evaluation, inclusion criteria, and rehabilitation metrics, as well as to optimize protocols and investigate the long-term effects. The review provides an overview of the current research methods and findings in immersive VR use. It offers insights into the field’s current approaches, serving as an initial step toward therapy standardization and addressing research standards’ current limitations.

### Supplementary Information

**Additional file 1: Table S1** Search strategy in the three databases: Scopus, PubMed, and IEEE Xplore.

## Data Availability

Not applicable.
