# Supplementary material for: Immersive VR for upper-extremity rehabilitation in patients with neurological disorders: a scoping review
Source: J Neuroeng Rehabil. 2024 May 11;21:75. doi: 10.1186/s12984-024-01367-0 (PMC11088157; doi:10.1186/s12984-024-01367-0)
Supplement: Supplementary file 1 — Additional file 1: Table S1 Search strategy in the three databases: Scopus, PubMed, and IEEE Xplore. [file 12984_2024_1367_MOESM1_ESM.docx]

# Additional file 1

**Table S1** | Search strategy in the three databases: Scopus, PubMed, and IEEE Xplore

| **Database** | **Search Query String** |
| --- | --- |
| *Scopus* | TITLE-ABS-KEY ( ("head mount*" OR "head-mount*" OR "hmd*" OR "hmd-based" OR "head-worn" OR "head worn" OR "headworn" OR "helmet-mounted display*" OR "visual display*" OR "headset*" OR "helmet*" OR "VR tool*" OR "display-based" OR "display based") AND ("vr" OR "ve" OR "virtual reality" OR "virtual game" OR "virtual environment" OR "VR environment" OR "immersive VR" OR "simulated 3D" OR "3D simulator" OR "3D game*" ) AND ( "upper limb*" OR "upper extremit*" OR "arm*" OR "forearm*" OR "hand*" OR "elbow*" OR "shoulder*" OR "wrist*" ) AND ( "rehabilitation*" OR "therap*" OR "treatment*" OR "intervention*") ) AND LANGUAGE(english) AND ( LIMIT-TO ( DOCTYPE,"ar" ) ) |
| *PubMed* | ("Smart Glasses"[MeSH Terms] OR "head mount*"[Title/Abstract] OR "head mount*"[Title/Abstract] OR "hmd"[Title/Abstract] OR "hmd based*"[Title/Abstract] OR "head-worn"[Title/Abstract] OR "head-worn"[Title/Abstract] OR "headworn"[Title/Abstract] OR "helmet mounted display*"[Title/Abstract] OR "visual display*"[Title/Abstract] OR "headset*"[Title/Abstract] OR "helmet*"[Title/Abstract] OR "VR tool"[Title/Abstract] OR "display-based"[Title/Abstract] OR "display-based"[Title/Abstract]) AND ("Virtual Reality"[MeSH Terms] OR "VR"[Title/Abstract] OR "VE"[Title/Abstract] OR "VR environment"[Title/Abstract] OR "immersive VR"[Title/Abstract] OR "simulated 3D"[Title/Abstract] OR "3D simulator"[Title/Abstract] OR "3d game*"[Title/Abstract]) AND ("Upper Extremity"[MeSH Terms] OR "Arm"[MeSH Terms] OR "upper limb*"[Title/Abstract] OR "upper extremit*"[Title/Abstract] OR "arm"[Title/Abstract] OR "arms"[Title/Abstract] OR "forearm*"[Title/Abstract] OR "hand*"[Title/Abstract] OR "elbow*"[Title/Abstract] OR "shoulder*"[Title/Abstract] OR "wrist*"[Title/Abstract]) AND ("Rehabilitation"[MeSH Terms] OR "Rehabilitation"[Title/Abstract] OR "therap*"[Title/Abstract] OR "treatment*"[Title/Abstract] OR "intervention*"[Title/Abstract]) AND "english"[Language] NOT (Clinical Conference) |
| *IEEE Xplore* | ("Index Terms":"head mount*" OR "Index Terms":"head-mount" OR "Index Terms":"HMD" OR "Index Terms":"hmd-based" OR "Index Terms":"head-worn" OR "Index Terms":"head worn" OR "Index Terms":"headworn" OR "Index Terms":"helmet-mounted display*" OR "Index Terms":"visual display*" OR "Index Terms":"headset*" OR "Index Terms":"helmet*" OR "Index Terms":"VR tool" OR "Index Terms":"VR tools" OR "Index Terms":"display-based" OR "Index Terms":"display based") AND ("Index Terms":"vr" OR "Index Terms":"ve" OR "Index Terms":"virtual reality" OR "Index Terms":"virtual game" OR "Index Terms":"virtual environment" OR "Index Terms":"VR environment" OR "Index Terms":"immersive VR" OR "Index Terms":"simulated 3D" OR "Index Terms":"3D simulator" OR "Index Terms":"3D game" OR "Index Terms":"3D games") AND ("Index Terms":"upper limb*" OR "Index Terms":"upper extremit*" OR "Index Terms":"arm" OR "Index Terms":"arms" OR "Index Terms":"forearm" OR "Index Terms":"forearms" OR "Index Terms":"hand" OR "Index Terms":"hands" OR "Index Terms":"elbow*" OR "Index Terms":"shoulder" OR "Index Terms":"wrist") AND ("Index Terms":"rehabilitation" OR "Index Terms":"rehabilitations" OR "Index Terms":"therapy" OR "Index Terms":"therapies" OR "Index Terms":"treatment" OR "Index Terms":"treatments" OR "Index Terms":"intervention" OR "Index Terms":"interventions") |
